# The impact of performance feedback on corporate ESG performance: Mediating role of environmental strategy

**DOI:** 10.1371/journal.pone.0298471

**Published:** 2024-03-07

**Authors:** Changman Ren, Xiaoxing Lin

**Affiliations:** 1 Department of Business Administration, Ningde Normal University, Ningde, China; 2 Department of Business Administration, Wuhan University of Technology, Wuhan, China; Universidad Central de Chile, CHILE

## Abstract

**Purpose:**

The purpose of this study is to investigate the impact of performance feedback (performance expectation surplus, performance expectation deficit) on corporate ESG performance, and this paper also to investigate the role of environmental strategy as a mechanism in the impact of enterprises’ performance feedback on corporate ESG performance.

**Design/Methodology/Approach:**

The study used data from 3679 companies listed on the Shanghai and Shenzhen stock exchanges for the period 2009–2021 and also measured the intensity of corporate environmental strategies through analysis. Finally, we used a fixed effects model to test the research hypothesis.

**Findings:**

This study shows that enterprise performance feedback positively affects corporate ESG performance and that environmental strategy plays a significant mechanistic role in enterprise performance feedback and corporate ESG performance. Overall, performance expectation surplus negatively affects ESG performance, performance expectation deficit positively affects ESG performance, and the mechanism of environmental strategy plays a significant role in performance expectation deficit and ESG performance.

**Practical implications:**

The results of this study can help enterprises establish a scientific environmental management system, strengthen the supervision of enterprise environmental management, and have certain reference significance for enterprises to speed up the implementation of environmental protection measures.

**Originality/Value:**

This study adds to the literature by describing corporate ESG performance using performance feedback theory and explaining the inherent role of enterprise performance feedback in corporate ESG performance utilizing environmental strategies.

## Introduction

Environmental change, pollution events, and corporate responsibility have all emerged in recent years, and stakeholders such as governments, the public, and businesses have been increasingly worried about ESG systems [1]. Because of its emphasis on integrated environmental, social, and governance development, ESG (environmental, social, and governance) has become a significant component in judging a company’s overall performance [2–5].

ESG has been discussed in academia for more than 30 years [2], and scholars have conducted a large number of studies on ESG, focusing on board executive characteristics [6–10], firm structure [11–13], and industry heterogeneity [14]. In addition, some scholars have also studied the relationship between financial performance and ESG. DasGupta finds that poor financial performance motivates firms to improve ESG actions and that ESG controversies play an active intermediary role in this process [15], And according to Schanzenbach and Sitcoff, admissible ESG investments in US fiduciary law must meet the criterion that the ESG investment will directly benefit the fiduciary by increasing risk adjustment [16].

It is evident that scholars have a well-informed understanding of ESG, but most of them are based on the level of non-financial performance of firms, and there are relatively few studies on how the enterprise’s financial performance affects corporate ESG. According to the Theory of corporate Behavior, due to limited rationality, organizations tend to make "success" or "failure" judgments based on their own performance compared to expected performance [17]. When performance is below expectations, it stimulates managers to search for problems and seek changes; when performance exceeds expectations, it indicates that the company has achieved the desired goals and managers tend to maintain the status quo [18–21]. Thus, the enterprise performance feedback is an important factor influencing management decisions [22,23].

According to the existing literature, the role of enterprise performance feedback in influencing management’s ESG behavior has not been unclear. And management’s ESG awareness and commitment is one of the key factors influencing this role. If management does not actively focus on ESG issues or lacks a clear commitment to ESG, performance feedback may be difficult to use in their decision-making. In addition, external stakeholder pressure is an important factor in the role of performance feedback. Stakeholders such as investors, customers, and regulators make demands on corporate ESG performance, and these demands may force management to pay more attention to ESG issues because they need to meet the expectations of these stakeholders to safeguard reputation and business.

Corporate environmental strategy refers to the practice of firms adhering to regulatory mandates or proactively adopting measures to mitigate the adverse environmental effects resulting from their operations. The focal point of corporate strategy research has shifted towards establishing a favorable relationship with the environment and achieving sustainable development [24]. Hence, the topic of corporate environmental strategy has garnered significant attention among scholars studying corporate strategy [25].

Can performance feedback (performance expectation surplus, performance expectation deficit) impact corporate ESG performance in the context of global efforts to battle pollution? Can corporate environmental policies play a mechanical role in the relationship between performance feedback and corporate ESG performance? To the above questions, this study brings the notions of performance expectation surplus and deficit into corporate ESG research, and the paper studies changes in corporate ESG performance under these settings. This research also studies the process of corporate environmental management, elucidating the intrinsic link between enterprise performance feedback and ESG performance.

The purpose of this study is to analyze the reference effect of enterprise performance feedback on ESG performance, and to provide thoughts for the decision-making of "performance feedback-ESG performance". To this end, this study examines the relationship between performance feedback, environmental strategy, and ESG performance using a double fixed-effects model and a mediated-effects model based on data from 3679 A-share listed companies in China. After that, use robust and endogeneity tests to ensure that the results are reliable.

The contributions of this study are as follows: First, this study fills a gap in existing research by incorporating performance expectation surpluses and deficits into the corporate ESG research system. Second, by investigating the role of environmental strategy as a mediator, it elucidates the relationship between enterprise performance feedback and ESG performance, emphasizing the importance of implementing environmental strategy. Third, this paper uses textual analysis to create a more objective environmental strategy indicator, which provides a beneficial reference for future research.

The remaining contents of this study are as follows: section 2 provides a detailed review of the literature and presents the research hypotheses; section 3 describes the sample selection, measurement of the variables, and construction of the model; section 4 prescribes the results of the statistical estimation; section 5 discusses the results; and finally, the conclusion is presented.

## Literature review and hypotheses development

### Corporate behavior theory

Performance feedback is derived from the theory of corporate behavior proposed by Cyert & March. The theory of corporate behavior suggests that performance that does not meet the organization’s preset level of expectations will drive the firm to actively implement problem-oriented search actions until performance is back on track. When actual performance is higher than the desired level, corporate decision makers tend to believe that the current strategy is effective and opt to maintain it [17]. In the theory of corporate behavior, the core concepts involved include organizational expectations, performance feedback, problem-based search [26]. Organizational expectations reflect the level at which the organization’s future performance should be reached or accepted, and they serve as the primary reference point for determining the success or failure of its strategic initiatives [17]. In existing studies, firms mainly set expectation levels based on two reference points (historical performance and industry performance) [27,28]. historical performance focuses on the internal part of the organization, comparing present performance to historical performance, which in turn directs future actions done by the organization. Industry performance, focuses on the outside of the organization, comparing the organization’s present performance to that of the industry in order to assess whether the firm takes strategic actions that drive or depart from the industry. Greve also suggests that organizational expectations influence performance evaluation and search behaviors [29]. Performance feedback is an important link between organizational expectations and problem search, and it covers both performance surplus and performance deficit [30]. In this study, we define expected performance deficit as when enterprises’ actual performance is lower than expected, and expected performance surplus as when enterprises’ actual performance is higher than expected. Problem-based search is one of the core concepts in the theory of corporate behavior. It is a simple process of finding solutions to problems driven by performance deficit [31]. This helps to explain various organizational behaviors and outcomes such as strategic change, innovation, organizational learning, etc. According to performance feedback, corporations can understand the gap between actual performance and the expected level. When there is a performance deficit, performance feedback is able to map the problem to the performance deficit, allowing the organization to quickly find a solution without the need to explore and research the root cause of the problem too much. Relevant studies have also shown that when actual enterprise performance is higher than performance expectations (expectation surplus), past successes reinforce managers’ confidence in current strategies and structures [32], making managers content with the status quo and reluctant to take risks associated with activities such as strategic change [33] and innovation [18]. In contrast, if the enterprise’s actual performance falls short of expectations (i.e., the expectation gap), managers believe the firm is failing and will be dissatisfied with the status quo. This circumstance prompts managers to undertake several risky decisions, including strategic changes [34], R&D innovations [18], mergers and acquisitions [35], and other actions.

### Performance feedback and ESG performance

ESG refers to a company’s quantitative performance in three non-financial dimensions: environmental, social, and corporate governance. Specifically, in the environmental dimension, companies need to take up the responsibility of minimizing the impact of their operations on environmental factors such as climate change, biodiversity, energy efficiency, water scarcity, pollution, deforestation, and waste management [36] through measures such as controlling pollution, investing in eco-efficient technologies, and developing environmentally responsible policies; In the social dimension, companies have the responsibility to protect the social-ecological systems in which they operate and to achieve comprehensive political, ethical, cultural, and ecological development by focusing on labour standards, gender equality and diversity, employee welfare, and community relations [37]; In contrast to the preceding two dimensions, the corporate governance dimension has previously attracted attention due to its close relevance to investors’ interests and has become an important means of resolving conflicts between management and shareholders, primarily involving corporate actions in the areas of internal control, risk management, information symmetry and transparency, business ethics, and shareholders’ rights [38]. In essence, ESG performance is an integral part of a firm’s high-quality development, and in particular, it can help firms access high-quality capital support [39] and thus help to promote their investment returns [3]. Attig et al. argued that a firm’s ESG performance can release positive non-financial information to the market signals [40], which can bring a positive effect to its credit rating as well as improve the availability of capital and further promote its sustainable development. As a result, ESG is a metric for measuring business sustainability performance [41].

First, consider the connection between performance feedback and the environment. corporations can strengthen their environmental efforts by raising environmental awareness and enhancing environmental quality with the help of environmental investments. Managers may be fired when the enterprise’s actual performance is below the target level, making it more challenging to effect a turnaround in the short term. Consequently, in order to support the company’s social function, managers must take specific actions to strengthen corporate legitimacy [42]. in turn, extends stakeholders’ tolerance for subpar enterprise performance. Studies in the field of environmental protection have shown that environmental performance has become a significant component of modern corporate legitimacy [43], and that firms can improve their regulatory legitimacy by investing more in the environment to make sure that their actions comply with environmental laws and regulations. At the same time, increasing environmental investment to strengthen pollution control, cleaner production, and the greening of the community can help improve a firm’s reputation for environmental compliance and help it develop a green image. Companies that invest more in environmental protection are more likely to be recognized and trusted by the public than those that invest less, and this boosts their perceived and regulatory legitimacy [44]. For green innovation, performance expectation surplus can cause managers to feel "rich and anxious" about innovation inertia, making them less willing to carry out innovative activities. On the contrary, the performance expectation deficit can make managers pay attention to green innovation. At this time, managers use the dual value effect of green innovation to overcome performance difficulties [45].

Second, consider the connection between performance feedback and CSR. There is a strong correlation between CSR and ESG. Many people often refer to corporate compliance with ESG standards as corporate social responsibility [46], and CSR is one of the pioneers of ESG [47]. Climate change, depletion of natural resources, poor working conditions, and negative corporate news have increased social expectations of corporate resources, and social responsibility [48]. There is an increasing emphasis on socially responsible activities, and companies are encouraged to take actions that address core social needs and create consistency between company operations and social values [49]. Therefore, CSR development and application generate what companies call shared value [47]. However, According to Ioannou and Serafeim, there is a need for greater concern over the many pressures that corporations encounter in relation to the disclosure of environmental, social, and governance factors [50]. Companies engage in social activities according to stakeholder expectations and utilize ESG disclosure as a tool to show that they are socially conscious [51]. They start monitoring improvements and modifications to their operations by associating particular performance with their social responsibility initiatives [52]. Companies can evaluate their environmental, social, and governance programs and generate shared benefits by engaging in corporate social responsibility (CSR).

Third, consider the connection between performance feedback and Corporate Governance. When the actual performance of an enterprise is higher than the desired performance, future expectations increase, and the likelihood of the enterprise failing to fulfill those expectations increases [53], which means that managers need to meet future expectations and carry more pressure to perform. In contrast, improving performance by reducing environmental governance costs or even violating emissions is easier to achieve than innovation activities [54]. Moreover, performance surpluses lead to managerial ego [50], which causes managers to focus more on the benefits of reducing environmental governance costs and ignore the harms [55].

The present study reveals a robust association between performance feedback and various dimensions of environmental protection, corporate social responsibility, and corporate governance. The concept of correlation encompasses both correlation and causation, with the establishment of causality being subject to certain limitations such as assumptions, interference from other variables, and the stability of time series data. Consequently, the criteria for establishing causation are more rigorous.

Based on these arguments, the following hypotheses are proposed:

H1: The performance expectation surplus has a negative correlation with corporate ESG performance.
H2: The performance expectation deficit has a positive correlation with corporate ESG performance.

### Environment strategy

Environmental strategy plays an important role in the implementation of environmental protection and environmental governance. Sharma and Vredenburg argues that a corporate environmental strategy is an action strategy that incorporates its environmental issues into the strategic decisions of corporate development [56], i.e., complying with rules and proactively taking environmental measures to reduce the negative impact of the company on the natural ecological environment in the daily production and operation process. Thus, environmental strategy is a form of strategy that aims to achieve sustainable corporate development by managing the "business-nature" interface [57]. Based on the above perspectives, the present study delineates environmental strategy as an organization’s deliberate choice to adhere to regulatory mandates or adopt proactive measures to execute ecological safeguarding practices, with the goal of reducing the negative environmental impact caused by its operations. Porter and Van der Linde suggest that environmental management, technological innovation, process improvement, and resource conservation are dynamically linked systems. They argue that firms should utilize environmental management as a corporate strategy to enhance competitiveness [58]. According to a review of the literature, various factors have a significant impact on the adoption of environmental initiatives. These concerns include environmental laws [59], corporation economic interests [60], stakeholder pressure [61], corporate resource level [62], and managers’ cognition [63].

Companies pay attention to environmental strategies and integrate environmental management into the whole process of their production and operation, which can help companies save intangible resources such as knowledge, human capital, and organizational culture [25] and enhance their learning ability, stakeholder integration, and green innovation [23]. Previous research has shown that implementing environmental strategies helps firms improve both their environmental and economic performance [64,65], and it is a win-win decision for firms to invest their resource capabilities in environmental practices [66,67].

The performance expectation surplus and deficit significantly affect corporate environmental strategy. Enterprises should explain the performance expectation gap in a reasonable manner and respond to concerns voiced by stakeholders such as shareholders and investors. Corporate environmental strategy is expensive [68], and good environmental performance implies higher environmental costs. Implementing a corporate environmental strategy can cement relationships with stakeholders by exposing them to the resources required to improve enterprise performance. The government acknowledges environmentally responsible corporations and gives them preferential funding and tax breaks [69]. When it comes to green transformation, it is inevitable for enterprises to pay attention to environmental issues and actively engage in environmental actions. Environmental strategy is critical for increasing enterprise financial performance, strengthening entrepreneurs’ image, and defining firms’ competitive advantage [70]. Suppose an enterprise’s actual performance is better than expected; decision-makers will pay less attention to risk strategies and suffer more stress from stakeholders to maintain the steady development of the enterprise [71]. They tend to operate conservatively and forgo those risky and costly activities. Therefore, corporations will make fewer efforts and investments in environmental strategies.

The implementation of environmental strategy improves the environmental, social and governance performance of corporations. Schaefer concluded through a case study approach. He argued that managers would strive to enhance economic and environmental performance [72], provided that enterprises incorporate environmental management into the strategic planning framework. Clarson et al. conducted empirical research on more than 100 listed companies, believing that environmental management positively correlates with economic performance [73]. Many academics believe that corporate efforts in pollution prevention and control, product oversight, end-of-pipe treatment, and sustainable development improve environmental performance [74–76]. Pollution prevention technologies can also improve corporations`production and environmental performance [77]. The existing research on the impact of environmental management practices on management innovation and environmental performance also proved that environmental management practices could effectively improve the environmental performance of corporations [68]. Finally, Corporations that develop forward-looking environmental strategies generally emphasize stakeholder integration, organizational learning, and green innovation, thus significantly improving their cost advantage, social reputation, and social performance. After researching eight dimensions of corporate social performance (community, corporate governance, diversity, employee relations, environment, employee rights, products, and others), Chen discovered that engagement in environmental issues and product safety could significantly translate into social performance [21]. Furthermore, Environmental governance and strategy may be viewed as severe challenges that require attention and resolution by enterprises that take a proactive environmental approach. Aragón-Correa and Sharma argue that enterprises have the capacity to use opportunities for growth by proactively addressing environmental concerns [78].

It is evident that when organizations receive performance feedback, they are motivated to modify their plans in order to enhance their ESG performance by implementing environmental policies. Finally, ESG is the consequence of performance feedback, which is obtained primarily through the application of environmental policies.

Based on these arguments, the following hypotheses are proposed:

H3: The mediating effect of environmental strategy is of considerable importance in relation to the performance expectations surplus and ESG performance.
H4: The mediating effect of environmental strategy is of considerable importance in relation to the performance expectations deficit and ESG performance.

## Methodology

### Sample

This study uses secondary data to analyze the connection between enterprise performance feedback and ESG performance for Chinese A-share listed companies from 2009 to 2021. After the outbreak of the U.S. subprime mortgage crisis in 2008, which had a serious impact on various industries around the world, most of the enterprises’ revenues decreased, which biased the construction of the performance feedback indicators of this study, so this study chose 2009–2021 as the sample time frame. The data for the appropriate years came from databases maintained by China Stock Market Accounting Research (CSMAR) and Wingo. From among the 3679 Chinese A-share listed businesses, we selected a research sample consisting of 30491 company-year observations due to the exclusion of firms with incomplete data, companies listed after 2012, companies issuing other forms of shares, and special treatment (ST) companies.

### Variable

This study selected variables and models based on literature analysis. Specifically, this study selects performance expectation surplus and performance expectation deficit as indicators of enterprise performance feedback, while environmental strategy is considered as an important factor affecting corporate ESG performance. In terms of model selection, this study adopts the fixed effect model for validation.

(1) Dependent variable. Referring to Wang et al., this study adopts the ESG score data from Hexun.com as an indicator for evaluating corporate ESG responsibility [79]. The score ranges from 1 to 100, which covers the score of overall corporate ESG responsibility disclosure as well as the treatment of different dimensions of ESG responsibility fulfillment performance. The score is based on social responsibility reports and financial reporting information from China Listed Companies, and it includes 13 secondary indicators and 37 tertiary indicators in five categories. Shareholder Responsibility accounts for 30% (10% profit, 3% reimbursement, 8% return, 4% innovation), Employee Responsibility accounts for 15% (5% performance, 5% safety, 5% employee care), Supplier, Customer, and Consumer Responsibility accounts for 15% (7% product quality, 3% after-sales service, 5% honesty and reciprocity), and Environmental Responsibility accounts for 20% (environmental protection awareness 2%, certification of an environmental management system 3%, investment in environmental protection 5%, sewage 5%, energy savings 5%), social responsibility 20% (10% income tax on total profit, 10% public welfare gift).
(2) Independent variables. According to the Theory of corporate Behavior, there are two main levels of performance expectations that managers use as a point of reference: historical expectations and social expectations [17]. We use historical expectations as a point of reference to compare the past performance of the target enterprise with its current performance [80]. On the other hand, enterprises use social expectations as a benchmark to assess the level of performance of other competitors within the same industry.

Thus, This study adopts Chrisman and Patel and takes the difference between the actual performance of the corporation and the performance expectation (P-A) to measure [81]. P is the actual performance of the corporation, and the commonly used performance measures are ROA and ROE. Since ROA accounts for the impact of both equity and debt funding, it is less affected by variations in financial leverage than other measures of a corporation’s success [18,21,82]. A is the performance expectation of the corporation, and the specific formula is as follows:

$$A_{i,t}={\alpha HA}_{i,t-1}+\left(1-\alpha \right){SA}_{i,t}$$ (1)

Where HA is the expected historical performance of company *i* as assessed by ROA in year *t*-1; SA is the average ROA in year t of companies in company *i* industry other than company *i*; and α is the representative weight, which typically ranges from 0.3 to 0.7. According to Chen, the linear fit of the model is highest when setting α to 0.6, so this study uses α = 0.6 as a criterion to calculate the difference between the actual performance P and the desired target A [21].

Referring to the study of Sengui and Obloj, a negative value of the difference between P and A indicates that the corporation failed to achieve the desired goal [83], i.e., the enterprise is in a performance expectation deficit (*GAPL*_*i*,*t*_). In this state, the data retains only the values where the difference is negative (*P*_*i*,*t*_–*A*_*i*,*t*_ < 0), and the rest of the values are set to 0. If the difference between *P* and *A* is positive, indicating that the enterprise achieved the desired goal, i.e., the enterprise is performance expectation surplus (*GAPS*_*i*,*t*_), the data retains the values where the difference is positive (*P*_*i*,*t*_–*A*_*i*,*t*_ ≥ 0) and the rest of the values are set to 0.

(3) Mediating variables. Referring to Wang et al., this study measures corporate environmental strategy by calculating the word frequency of environmental keywords included in the text of CSR reports through detailed descriptions of corporate environmental behavior [84]. Based on Sapir-Whorf’s assumption, the frequently occurring words in this text are in the center category of individual cognition, reflecting what individuals are most concerned about. Quantifying the words and phrases containing environmental topics in CSR report texts can capture the extent of corporate concern and investment in the environment. word frequency methods have emerged in the field of business management research in recent years, and Weber pointed out that word frequency represents the degree of importance given to words in a text [85]. This study uses a lexicon-based "seed word + Word2vec" similarity word expansion method to construct environmental strategy indicators to capture the degree of corporate concern and commitment to environmental issues by processing words and phrases related to environmental issues in social responsibility reports and combining them with the Word2vec neural network word vector model proposed by Wang et al. and short et al. [84,86] As the most commonly used method for constructing text metrics, the lexicon method has received widespread attention because of its comprehensibility and ease of replication [87,88]. The specific steps of indicator construction are as follows: first, The seed word for this study is "environmental protection." this is due to the fact that, although environmental protection issues have long been the focus of attention in Western countries, the Chinese government has recently continued to pay attention to green environment, carbon emissions, carbon neutrality, and has introduced many specific measures on environmental protection. Second, This research use Word2vec modeling for similarity word extend based on corporate environmental strategy seed words. The model is based on the linguistic notion of defining similarity based on cosine distance, i.e., semantically comparable words with similar nearby words [89]. The deep learning training procedure converts the words into real-valued vectors, creating a word vector space. Lastly, 50 terms that are comparable to the seed words and have a similarity score higher than 0.5 are selected and will be utilized to investigate the problems related to corporate environmental strategy (Table 1). Finally, We entered fifty similar words into the Wingo database, and the degree of attention to corporate environmental strategy was measured by calculating the percentage of these similar words in the CSR report. Compared to using the total number of words to measure word frequency, this method can effectively avoid the influence of document size on the test results by calculating the percentage of the total number of environmental strategy words in the total number of words in the text of the social responsibility report [74].

**Table 1:** Keywords on CES in the CSR reports.

Category	Keywords
**Seed word**	Environmental Protection
**Similar words**	Environmental management, climate change, environmental quality, energy-saving and consumption reduction, sustainable, green environmental standards, pollution, healthy, warming, resource conservation, environmental pollution, energy, energy-saving, energy management, ecological environment, natural, low-carbon, clean, ecological, harmonious, improvement, water resources, green ecology, comprehensive management, natural resources, waste, emission reduction, hazardous substance, atmospheric environment, waste reduction, climate, cleanness, emissions, sustainability, cycle, recycling, waste discharge, greenhouse, negative carbon, safety incident, energy conservation, discharge, utilization, governance, pollution control, natural gas, natural ecology, environment standards, consumption reduction, climate warming

(4) Control variables. Environmental strategy and ESG performance require firms’ continuous attention and investment. Big-size companies have more resources to focus on and implement ESG strategies. Low-leverage and older companies may focus more on corporate stability, while high-leverage and younger companies will be more adventurous. Fast-growing companies may face resource pressures that affect ESG strategy implementation. Larger boards may be more inclusive, but decision-making processes may be complex. Therefore, the following variables were controlled in this study. firms’ life cycles, and firms’ operating conditions have an impact on environmental strategy and ESG performance, this study controls for the following variables: size, measured as the natural logarithm of the firm’s total assets at the end of the year; Lev, measured as the ratio of total liabilities to total assets; Growth, the growth rate of operating revenue; Board, the number of board members, and age (Table 2).

**Table 2:** The defination of control variables.

Variables	Measurement	References
**Size**	Natural logarithm of total assets	[18,28,38,84,86,87]
**Lev**	Total liabilities/total assets	
**Growth**	sales growth rate	
**Board**	Number of Board menbers	
**Age**	Age of establishment of enterprise	

### Models

The estimation methods for panel data are divided into various types, and the appropriate analytical model is determined prior to data analysis. The regression model applied to the panel data is determined by the F and Hausman tests (Table 3). The F-test results show (p<0.001) that the fixed effects model outperforms the mixed effects model, while the Hausman test results (p<0.001) once again tell us that the fixed effects model again outperforms the random effects model. Fixed effects modeling has several advantages when dealing with panel data and the long-run relationship of variables. It enables more accurate estimation of the relationship between variables by taking into account individual and time-fixed effects while dealing with unstable data, avoiding the problem of spurious regressions, and providing reliable results. Furthermore, the model can evaluate panel data across time and among individuals, which facilitates a thorough investigation of the correlations between the variables.

**Table 3:** Model test results.

HypothesesH0	F test P	Hausman test P
Mixed effects are better than fixed effects	0.000	-
Random effects are superior to fixed effects	-	0.000

After the F-test and Hausman test, it was decided that a two-way fixed-effects model was needed for this study (Table 3). so this study used firm (i) and year (t) fixed effects. This study builds a regression model (2) to test hypotheses H1 and H2, adds environmental strategy factors to model (2), then builds regression models (3) and (4) to test hypotheses H3 and H4.

$$ESG_{i,t}=\beta_{0}+\beta_{1}GAPL_{i,t}+\beta_{2}GAPS_{i,t}+\beta_{3}Control_{i,t}+\varepsilon_{I,t}$$ (2)

$$CES_{i,t}=\beta_{0}+\beta_{1}GAPL_{i,t}+\beta_{2}GAPS_{i,t}+\beta_{3}Control_{i,t}+\varepsilon_{I,t}$$ (3)

$$ESG_{i,t}=\beta_{0}+\beta_{1}GAPL_{i,t}+\beta_{2}GAPS_{i,t}+\beta_{3}CES_{i,t}+\beta_{4}Control_{i,t}+\varepsilon_{I,t}$$ (4)

where GAPL is the performance expectation deficit, GAPS is the performance expectation surplus, CES is the corporate environmental strategy, controls are the aforementioned control variables, and *ε* is the random error term in the model.

## Empirical results

### Descriptive statistics

Table 4 demonstrates the results of descriptive statistical tests for the main variables. The study shows that the mean value of corporate ESG performance is 72.873, indicating that the overall ESG performance of Chinese A-share listed companies is good. The enterprise performance expectation surplus has a mean value of 0.014 and a standard deviation of 0.033, suggesting that there exist variations in the performance expectation surplus among various enterprises. The observed enterprise performance expectation deficit of -0.018, along with a standard deviation of 0.041, suggests the presence of variations in the performance expectation deficit across different enterprises. Furthermore, the average value of corporate environmental strategy is 0.002, meaning that 0.2% of the words in CSR reports deal with environmental issues. The tiny amount of data suggests that corporations don’t focus as much on environmental issues in their social responsibility reports. Nonetheless, this data still offers some insight into the environmental plans of corporations, aiding in our comprehension of their actions and perspectives about the environment.

**Table 4:** Descriptive statistics.

Variable	Obs	Mean	Std. Dev.	Min	Max
**ESG**	30491	72.906	5.439	56.05	84.26
***GAPS***_***i*,*t***_	30491	.014	.033	0	.212
***GAPL***_***i*,*t***_	30491	-.018	.041	-.272	0
***CES***_***i*,*t***_	30491	.002	.002	.000	.009
**Size**	30491	22.181	1.308	19.576	26.229
**Growth**	30491	.399	1.093	-.679	7.950
**Lev**	30491	.434	.209	.055	.949
**Board**	30491	6.580	3.878	1	14
**Age**	30491	10.389	7.209	1	27

These descriptive statistics are relevant for our study. First, they give us thorough details on the variables and their distribution, which aids in our comprehension of the features and variations of the sample. Second, they support our efforts to determine whether the results are statistically significant, representative of the sample, and the validity and reliability of the regression analysis.

### Correlation analysis

The correlation analysis and multicollinearity between the main variables are shown in Table 5. The results show that the correlation coefficient between corporate ESG performance and enterprise performance expectation surplus is -0.22, which is significant at the 1% level, and the correlation coefficient between corporate ESG performance and enterprise performance expectation deficit is 0.11, which is significant at the 1% level, confirming hypotheses H1 and H2.There is a strong link between environmental strategy, company ESG performance, and enterprise performance expectation deficit. Further VIF value calculations revealed that all of the VIF values were less than 5, indicating that the variables were not multicollinear.

**Table 5:** Correlation matrix.

Variable	ESG	*GAPS*_*i*,*t*-1_	*GAPL*_*i*,*t*-1_	*CES*_*i*,*t*_	Size	Growth	Lev	Board	Age	VIF
**ESG**	1									
***GAPS***_***i*,*t***_	-0.22 ***	1								1.09
***GAPL***_***i*,*t***_	0.11 ***	0.18 ***	1							1.09
***CES***_***i*,*t***_	0.11 ***	0.01	0.03 ***	1						1.07
**Size**	0.25 ***	-0.09 ***	0.08 ***	0.24 ***	1					1.51
**Growth**	0.01	0.03 ***	0.04 ***	-0.06 ***	0.02 ***	1				1.02
**Lev**	-0.09 ***	0.03 ***	-0.10 ***	0.07 ***	0.42 ***	0.08 ***	1			1.33
**Board**	0.04 ***	0.01	0.01	0.01	0.22 ***	-0.04 ***	0.09 ***	1		1.06
**Age**	-0.07 ***	0.11 ***	-0.04 ***	-0.06 ***	0.33 ***	0.07 ***	0.32 ***	0.05 ***	1	1.22

Note: *, **, *** represent significant at the 10%, 5%, and 1% levels, respectively.

### Regession results

To guarantee the consistency and validity of the model estimations, the primary variables in this study area are winsorized at the 1% level before to completing the empirical analysis [90].

This study uses a two-way fixed effects model to examine the relationship between performance expectation surplus, performance expectation deficit and corporate ESG performance. Table 6 indicating that the regression coefficient of performance expectation surplus is significantly negative (β = -0.235, P <0.01), and the regression coefficient of performance expectation deficit is very positive (β = 0.202, P <0.05). It can be concluded that when the actual performance of enterprises is worse than expected, the ESG performance is more likely to improve with time; on the contrary, when the actual performance of enterprises is better than expected, the ESG performance is more likely to decline. Thus, H1 and H2 proposed in this study are supported.

**Table 6:** Regression results.

	ESG
***GAPS***_***i*,*t***_	-21.86*** (-29.22)
***GAPL***_***i*,*t***_	5.69*** (9.47)
**Size**	1.26*** (24.46)
**Growth**	-0.04 (-1.57)
**Lev**	-4.74*** (-22.59)
**Board**	-0.10*** (-3.50)
**Age**	0.15 (0.69)
**_Cons**	49.07*** (38.40)
**F**	128.43***
**Adj. *R*** ^**2**^	0.08

Note: *, **, *** represent significant at the 10%, 5%, and 1% levels, respectively.

### Robust test

This study employs performance feedback that is one period lagged, the substitution of ESG variables, and double difference method for robust testing to assess the hypothesis testing. First, The effect of performance feedback on corporate ESG performance is measured in this study using a one-period lag to performance expectation surplus and performance expectation deficit. Second, The ESG performance of the corporation is examined for robustness using the Huazheng’s index. Because of its proximity to the Chinese market, broad coverage, and high timeliness, Huazheng’s ESG rating data is frequently used in academics [91]. huazheng’s ESG index classifies companies into 9 levels, C, CC, CCC, B, BB, BBB, A, AA, AAA, from low to high, assigns 1,2 …8,9‥8,9 (Table 7). Finally, considering that the implementation of the 《New Environmental Protection Law》 in 2015 has the potential to bring exogenous shocks to the hypothesized results, this study employs a double-difference model to control for this. Specifically, the effect of the implementation of the《New Environmental Protection Law》 on enterprises’ ESG performance is examined using the period before the implementation of the New Environmental Protection Law as the control group and the period after the implementation as the experimental group. The model is:

$$ESG_{i,t}=\beta_{0}+\beta_{1}Tgapl_{i,t}\times Post_{i,t}+\beta_{2}Control_{i,t}+\varepsilon_{I,t}$$ (5)

$$ESG_{i,t}=\beta_{0}+\beta_{1}Tgaps_{i,t}\times Post_{i,t}+\beta_{2}Control_{i,t}+\varepsilon_{I,t}$$ (6)

$$ESG_{i,t}=\beta_{0}+\beta_{1}Tgapl_{i,t}\times Post_{i,t}+\beta_{2}Tgaps_{i,t}\times Post_{i,t}+\beta_{3}Control_{i,t}+\varepsilon_{I,t}$$ (7)

**Table 7:** Robust test.

	(1) *ESG*_*i*,*t*_	(2) *ESG*_*i*,*t*_
***GAPS***_***i*,*t*-1**_	-12.05*** (-14.85)	
***GAPL***_***i*,*t*-1**_	20.12** (31.32)	
***GAPS***_***i*,*t***_		-4.28*** (-27.04)
***GAPL***_***i*,*t***_		1.05** (8.3)
**Size**	1.28*** (22.79)	0.25*** (22.99)
**Growth**	-0.06** (-2.43)	-0.01 (-1.17)
**Lev**	-4.12*** (-18.15)	-0.93*** (-2.08)
**Board**	-0.11*** (-3.65)	-0.02*** (-2.9)
**Age**	0.21 (1.00)	0.01 (0.17)
**_Cons**	46.60*** (29.62)	-0.62*** (-2.3)
**F**	130.52***	110.19***
**Adj. *R*** ^**2**^	0.07	0.124

Note: *, **, *** represent significant at the 10%, 5%, and 1% levels, respectively.

Tgapl in the model denotes the sample firms’ performance expectation surplus throughout the observation period, with a value of 1 if it belongs to the high performance expectation surplus and 0 otherwise. Tgaps denotes the firm’s performance expectation fallout during the observation period, with a value of 1 if it belongs to the high performance expectation fallout and 0 otherwise. Post is a dummy variable that has the value 1 in the year of the 《new Environmental Protection Law》 implementation and future years and 0 in all other years. The analytical results reveal that the 《new Environmental Protection Law》 did not introduce exogenous shocks into the hypothesis test of our study, indicating that our hypothesis test is valid (Table 8). Overall, The results of the above robust tests are all consistent with the prior results, implying that the findings of this study are reliable.

**Table 8:** DID test.

	Model5 *ESG*_*i*,*t*_	Model6 *ESG*_*i*,*t*_	Model7 *ESG*_*i*,*t*_
***Tgaps***_***i*,*t***_	-0.681 (-0.76)		-0.713 (-0.74)
***Tgapl***_***i*,*t***_		-0.128 (-0.17)	0.126 (0.15)
**Size**	1.494*** (25.31)	1.495*** (25.32)	1.494*** (25.31)
**Growth**	-0.076*** (-2.84)	-0.076** (-2.84)	-0.076*** (-2.84)
**Lev**	-5.393*** (-22.47)	-5.389*** (-22.45)	-5.394*** (-22.47)
**Board**	-0.089*** (-2.85)	-0.09*** (-2.85)	-0.089*** (-2.85)
**Age**	-0.000 (-0.02)	-0.005 (-0.02)	-0.005 (-0.02)
**_Cons**	43.164*** (14.29)	42.808*** (14.21)	43.105*** (14.20)
**F**	154.16	154.11	132.13
**Adj. *R*** ^**2**^	0.59	0.59	0.59

Note: *, **, *** represent significant at the 10%, 5%, and 1% levels, respectively.

### Endogenneity analysis

Since enterprise performance feedback is substantially endogenous to the firm’s own characteristics, we use the instrumental variable approach and the propensity score method to account for it, even if it influences corporations’ ESG performance. First, This study selects a lagged performance feedback as a instrumental variable and uses a two-stage least squares method to mitigate the problem. The instrumental variables approach is a statistical strategy for solving endogeneity problems that uses one or more exogenous variables (i.e., variables that are not impacted by endogeneity) as instruments to estimate the parameters of endogenous variables. This study first finds the endogeneity problem, then chooses the right instrumental variables, does a first-stage regression to get the predicted values of the endogenous variables, then does a second-stage regression to get the updated parameter estimates, and finally checks that the instrumental variables are valid. The results show that performance expectation surplus, performance expectation deficit and corporations’ ESG are expected to conform to the hypotheses (Table 9). Second, this study uses the PSM to deal with endogeneity. Based on the difference between P and A as a benchmark, we divide the performance feedback into two groups (positive and negative) and assign the values 1 and 0. After that, we use the nearest neighbor matching method to find the treatment and control groups. Finally, after passing the balance test, regression analysis is performed on the matched samples, and the results show that our model is robust (Table 10).

**Table 9:** 2SLS test.

	First stage *GAPS*_*i*,*t*-1_	First stage *GAPL*_*i*,*t*-1_	2SLS *ESG*_*i*,*t*_
***GAPS***_***i*,*t*-1**_	0.971*** (-39.85)		
***GAPL***_***i*,*t*-1**_		0.975*** (43.85)	
***GAPS***_***i*,*t***_			-33.72*** (-10.17)
***GAPL***_***i*,*t***_			16.14*** (6.62)
**Size**	-0.01*** (-20.06)	0.006*** (11.99)	1.0865*** (16.82)
**Growth**	0.002*** (12.06)	0.003*** (10.74)	-0.038 (-1.49)
**Lev**	0.01** (5.68)	-0.066*** (-30.54)	-3.875*** (-13.73)
**Board**	-0.001** (-2.11)	-0.000 (-0.01)	-0.106*** (-3.67)
**Age**	0.004** (2.35)	-0.005** (-2.27)	0.262 (1.24)
**_Cons**	0.17*** (15.38)	-0.09 (-6.78)	
**Underidentification test** **Weak identification test**			1368.59*** 720.59***
**Adj. *R*** ^**2**^	0.09	0.12	0.07

Note: *, **, *** represent significant at the 10%, 5%, and 1% levels, respectively.

**Table 10:** PSM test.

	Before PSM *ESG*_*i*,*t*_	After PSM *ESG*_*i*,*t*_
***GAPS***_***i*,*t***_	-21.86*** (-29.22)	-21.58*** (-20.07)
***GAPL***_***i*,*t***_	5.69*** (9.47)	5.08*** (5.97)
**Size**	1.26*** (24.46)	1.36*** (18.61)
**Growth**	-0.04 (-1.57)	-0.05 (-1.40)
**Lev**	-4.74*** (-22.59)	-4.67*** (-15.77)
**Board**	-0.10*** (-3.50)	-0.08** (-2.01)
**Age**	0.15 (0.69)	-0.15 (0.57)
**_Cons**	49.07*** (38.40)	46.56*** (26.02)
**F**	128.43***	66.53***
**Adj. *R*** ^**2**^	0.08	0.07

Note: *, **, *** represent significant at the 10%, 5%, and 1% levels, respectively.

### Mechanism effect analysis

This study aims to test the mediating effect of corporate environmental strategy. For this purpose, we used Baron and Kenny’s [92] stepwise test of mediation effects and regression analysis of Eqs (3) and (4). According to the findings in Table 11, the coefficient of performance expectation surplus in column (1) is significantly negative (= -33.13, p<0.01), whereas the coefficient of performance expectation surplus in column (2) is insignificant (= 0.0003, p>0.1). It suggests that corporate environmental strategy does not mediate effect in the relationship between performance expectation surplus and corporations’ ESG performance. Thus, hypothesis H3 is invalid. However, the coefficient of performance expectation deficit in column (1) is significantly positive (= 13.56, p<0.01), the coefficient of performance expectation deficit in column (2) is also significantly positive (= 0.0009, p<0.01), and the coefficient of performance expectation deficit in column (3) is significantly positive (= 13.38, p<0.01) and smaller than the coefficient of 13.56 in column (1). The environmental strategy coefficient is significant (= 193.83, p<0.01), indicating that the environmental strategy plays a partially mediating function in the performance expectation deficit and ESG performance, implying that hypothesis H4 is correct. The results of this study point out the relationship between performance expectation deficit, corporate environmental strategy and corporate ESG performance. When enterprises face a performance expectation deficit, managers are more inclined to seek changes and adopt environmental strategies to reduce costs and increase efficiency through improving ESG performance. However, when enterprises are in performance expectation surplus, their environmental strategies do not play a mediating role, which may be due to the fact that enterprises focus more on short-term economic benefits and ignore the impact of environmental strategies on ESG performance. These findings highlight the mediating effect of performance expectations for the relationship between ESG performance and a corporation’s environmental strategy, and the fact that the role of environmental strategy varies across performance expectations. These findings provide important guidance for corporations in developing strategies and managing performance.

**Table 11:** Mediating effect of CSR.

	(1) *ESG*_*i*,*t*_	(2) *CES*_*i*,*t*_	(3) *ESG*_*i*,*t*_
***GAPS***_***i*,*t***_	-33.13*** (-37.20)	0.0003 (1.38)	-33.2*** (-37.35)
***GAPL***_***i*,*t***_	13.56*** (18.88)	0.0009*** (4.12)	13.38*** (18.66)
***CES***_***i*,*t***_			193.83*** (10.74)
**Control**	Yes	Yes	Yes
**_Cons**	147.03*** (8.93)	-0.25*** (-47.72)	195.26*** (11.46)
**F**	725.9***	578.76***	660.47***
**Adj. *R*** ^**2**^	0.159	0.132	0.163

Note: *, **, *** represent significant at the 10%, 5%, and 1% levels, respectively.

### Heterogeneity analysis

The mechanism test reveals that environmental strategy is one of the key elements in ESG performance, and companies should pay attention to and continuously improve their environmental strategy to ensure that they achieve positive impacts and results in ESG. However, in addition to focusing on environmental strategy as an internal factor, we should also focus on the impact of external factors on the relationship between performance feedback and ESG performance. External influences can provide key information about a company’s ESG performance, which can help a company better meet the expectations of investors, shareholders, regulations, and market competition, while managing potential ESG risks and contributing to the company’s success in sustainability and long-term value creation.

Therefore, this study also examines the role of external factors such as government environmental regulations, industry competition, and external regulation in performance feedback and corporate ESG performance. As a result, a heterogeneous study of performance feedback and corporate ESG performance is conducted, with a high pollution industry dummy variable (Highpo), a competitive market industry dummy variable (Highind), and a media attention dummy variable (Highnews) included to interpret the impact of performance feedback on corporate ESG performance in various external contexts (Table 12).

**Table 12:** Heterogeneity test.

	*ESG*_*i*,*t*_ HighPo	*ESG*_*i*,*t*_ LowPo	*ESG*_*i*,*t*_ Highind	*ESG*_*i*,*t*_ Lowind	*ESG*_*i*,*t*_ Highnews	*ESG*_*i*,*t*_ Lownews
***GAPS***_***i*,*t***_	-17.53*** (-12.63)	-23.32*** (-25.82)	-23.71*** (-16.52)	-20.09*** (-21.90)	-16.46*** (-10.75)	-22.98*** (-25.27)
***GAPL***_***i*,*t***_	3.42*** (2.68)	6.35*** (9.29)	7.17*** (6.31)	3.62* (4.82)	3.99** (3.29)	5.74*** (7.84)
**Size**	1.23*** (11.07)	1.24*** (20.22)	1.10*** (10.53)	1.31*** (20.16)	1.48*** (12.59)	1.24*** (20.11)
**Growth**	-0.09 (-1.58)	-0.02 (-0.80)	-0.15*** (-3.03)	-0.01 (-0.08)	-0.03 (-0.73)	-0.02 (-1.00)
**Lev**	-4.98*** (-11.86)	-4.59*** (-18.15)	-4.34*** (-10.74)	-4.87*** (-18.41)	-5.73*** (-12.22)	-4.32*** (-17.15)
**Board**	-0.11* (-1.93)	-0.09*** (-2.92)	0.01 (0.30)	-0.14** (-4.03)	-0.15*** (-2.80)	-0.06***(-1.90)
**Age**	-0.85 (-1.48)	0.28 (1.30)	0.48 (0.98)	-0.19 (-0.78)	0.19 (0.34)	-0.04 (0.18)
**_Cons**	53.78*** (16.02)	48.78*** (33.78)	50.06*** (18.26)	49.47*** (31.36)	43.67*** (12.79)	49.44*** (34.18)
**F**	28.8***	95.15***	37.23	77.27	26.24***	93.11***
**Adj. *R*** ^**2**^	0.04	0.03	0.08	0.11	0.109	0.017

The results in Table 10 show compliance with our predictions, industry competitive pressure positively affects performance expectation deficit, and environmental regulation and media attention positively affects performance expectation surplus.

## Discussion

This study’s analysis provides substantial evidence for testing hypotheses. There is a negative correlation between corporation ESG performance and performance expectation surplus, a positive correlation between performance expectation deficit and ESG performance, and a mediating role for environmental strategy between performance expectation deficit and ESG performance. The only inconsistency with our hypothesis is the insignificant mediating role of environmental strategies in the performance expectation surplus and corporate ESG.

Performance expectation deficit positively affects corporate ESG performance, while performance expectation surplus negatively affects corporate ESG performance. **First,** The performance expectation deficit is a deterministic risk [93] that stimulates entrepreneurial risk-taking and drives strategic change in corporations [94]. When corporations face performance expectation insufficient, managers adjust resource allocation and increase R&D investment [95,96] to improve innovation efficiency [97] and realize increased corporate performance [18,20,21]. In addition, to reverse the disadvantage, firms will intensify their innovative behavior [98], resulting in a performance expectation deficit that will enhance green innovation dynamics [45]. In addition to reducing environmental pollution and energy consumption to achieve environmental performance [99], green innovation also promotes economic performance through green product development [100]. **Second,** The performance expectation surplus is too high, meaning that it has reached its stated goals, and decision-makers are more likely to stick to old ways of managing the organization and pay less attention to risk strategies. When an enterprise’s actual performance is better than its desired goals, it may raise hopes for the future and make it more likely that those expectations will come true [53,101]. To keep the gains that have already been earned safe, company leaders may decide to be cautious and not use environmental inputs that have long investment cycles and high risks. Another thing is that a corporation’s environmental inputs might not directly affect its economic performance [102]. Instead, they might make corporate operations riskier because of the high costs of environmental protection facilities, technological research and development, and new ideas [103]. **Third,** there are certainly similar studies that contradict the results of this study. When actual performance falls short of the intended target, firms with limited resources reduce redundant resources and experience increased pressure due to resource constraints. To deal with this dilemma, managers may boost market behaviors while decreasing environmental governance behaviors, which waste resources and make it harder to improve performance fast [104]. This view differs somewhat from the results of this study. The viewpoint of this study emphasizes that the performance expectation deficit can promote sustainable development, i.e., firms are more inclined to adopt long-term strategic planning, including initiatives such as increasing environmental governance and optimizing resource allocation, to achieve the goal of sustainable development after becoming aware of the performance expectation deficit. In addition, performance feedback in this study focuses on performance expectation surplus and performance expectation deficit, while other studies may only focus on the magnitude of performance feedback. Environmental protection has become a global theme, and increased investment in environmental protection by enterprises can fulfill the requirements of environmental protection laws and regulations [105], which helps to enhance the legitimacy of enterprises. A firm’s green image is more likely to be recognized by the public, thus increasing the firm’s normative legitimacy and perceived legitimacy [44].

Environmental strategy plays a mediating role between the performance expectation deficit and corporate ESG performance, while it does not play a significant role between the performance expectation surplus and corporate ESG performance. **First,** The performance expectation deficit not only shows the shrinking of the firm’s competitive advantage and the failure of the established strategy, but also triggers stronger stakeholder governance pressures and legitimacy crises. These internal and external pressures force organizations to rethink their strategic activities and business directions in order to solve organizational problems [106]. Environmental strategy is crucial for a firm’s long-term competitive advantage [70] and important for enterprises to expand their knowledge and technological capabilities [25]. Bhatia’s study shows that a proactive environmental strategy is an important antecedent to green process innovation [107]. In addition, by implementing green product innovation and other driving environmental strategies, enterprises can help them reshape the market space, break homogenized market competition to achieve profitability, reduce production costs, and mitigate performance decline [89]. Environmental strategies are voluntary environmental protection initiatives implemented by corporations that go beyond traditional environmental rules and codes of conduct. Roxas and Coetzer also stated that managers are more likely to adopt positive attitudes toward environmental concerns when they believe the institutional context is supportive of the firm’s environmental management methods [108]. **Second**, Enterprise performance expectation surpluses may not affect environmental strategy because managers reduce risky strategic activities to avoid losses. Managers ascribe successful performance to the soundness of their strategic decisions, and managers build strategic self-confidence, which might limit an organization’s willingness to take risks. Successful experiences can boost managers’ confidence and make them less willing to make strategic changes [32]. When firms have a performance expectation excess, they are under pressure from stakeholders such as shareholders and creditors to maintain company stability [71]. Furthermore, an enterprise with a performance expectation surplus indicates that its resources and internal strategy are well aligned, and change may entail resource reallocation, which may result in lower returns than under the current arrangement [109]. **Third,** Although this study indicates that environmental strategy has a favorable impact on corporate ESG performance, it is unclear whether the strategy is implemented proactively by firms or passively by external constraints. According to Sharmas and Vredenbur categorization, environmental strategies can be divided into reactive and forward-looking types [56]. A forward-looking environmental strategy is one in which a company takes measures before environmental problems become apparent and prepares for force majeure factors. Compared to the reactive inputs of a proactive environmental strategy, a forward-looking environmental strategy is more likely to translate into a sustainable competitive advantage in product development and production. Enterprises can promote environmental strategies by proactively implementing activities such as green product innovation to reshape market space, break competition in homogenized markets, and achieve profitability. Several enterprises have successfully adopted these environmental strategies with positive results. For example, Ford Motor Company has improved its ESG performance and won market recognition through environmentally friendly production processes and the introduction of environmentally friendly models. Orsted has achieved its emissions reduction targets and improved its ESG performance and share price performance by vigorously developing and utilizing renewable energy sources such as wind power. Future research could further explore the impact of different types of environmental strategies on ESG performance, which would help to provide more theoretical support and guidance to companies.

This study also examines the role of factors such as government environmental regulation, industry competition and external regulation in the relationship between performance feedback and corporations’ ESG performance. Government environmental regulations and policies are an important means of promoting corporate concern for environmental issues. When governments strengthen environmental regulations, enterprises face stricter environmental regulation and greater environmental risks, and therefore need to pay more attention to environmental management and adopt environmentally friendly production methods and behaviors. As an important external regulator, media scrutiny can lead companies to pay more attention to environmental issues and adopt environmentally friendly measures. When enterprises are in a performance expectation surplus, the media may report on their positive economic, environmental, and social initiatives, thereby enhancing the firm’s reputation and incentivizing other firms to follow suit. Under a performance expectations surplus, highly polluting industries and high media attention force firms to invest more in innovation and environmental protection and to pay more attention to environmental performance. On the other hand, competition in the industry has an impact on falling performance expectations. In a competitive market environment, firms need to continuously improve their competitiveness to gain market share, which may prompt them to adopt more environmentally friendly production methods and behaviors to demonstrate their social responsibility and sustainability. At the same time, competition in the industry may also lead companies to adopt more proactive environmental measures to build a favorable corporate image and attract more consumers and investors. In this case, the pressure on managers leads to an increased risk appetite [110] and a tendency to opt for rapid corporate change to alleviate competitive pressures, with a consequent increase in environmental investment.

## Conclusion

Based on corporate behavior theory, this study investigates the influential relationship between performance feedback and corporate ESG performance, and the mediating effect of corporate environmental strategy. The results show that, First, enterprise performance expectation deficit promotes ESG performance. Meanwhile, enterprise performance expectations surplus reduce their ESG performance. Second, a corporation’s environmental strategy partially mediates the relationship between the performance expectation deficit and the corporation’s ESG performance. The mediating effect of corporations’ environmental strategies on performance expectations sirplus is not significant. Third, The heterogeneity results show that industry competition promotes the influence relationship between performance expectation deficit and corporations’ ESG performance, while government environmental regulation and media attention play a positive role in performance expectation surplus and corporations’ ESG performance.

This study investigates the importance of enterprises’ relative performance levels in environmental protection, fills a gap in the focus on the relative level of enterprise performance, and offers a new perspective for future relevant research. Meanwhile, from the standpoint of environmental protection, this study expands the area of research on corporate non-market behavior and enhances understanding of corporate performance feedback affecting ESG performance. Furthermore, this analysis demonstrates the importance of environmental strategy in explaining the inherent link between enterprise performance deficit and ESG performance, giving a new route for corporate environmental strategy research.

Based on the findings of this study, this study makes the following recommendations: **First,** Companies must build a scientific environmental management system by creating defined ESG objectives, linking them to managerial performance, reviewing and evaluating them on a regular basis, and strengthening ESG awareness and skills training. However, the implementation process may confront problems such as data collection and reporting difficulties and short-term performance constraints, necessitating a major investment of money and time as well as consideration of long-term ESG strategic planning. **Second,** Strengthening the oversight role of corporate environmental management requires enterprises to establish a dedicated internal audit department or team, develop an environmental risk management plan, and conduct regular assessments and audits of environmental inputs. However, these initiatives require a significant investment of resources, and enterprises also need to cope with the uncertainty and complexity of environmental risks. **Again,** The Government can encourage enterprises to fulfill their environmental protection responsibilities by providing policy measures such as financial subsidies and tax incentives, in addition to strengthening the supervision and management of enterprises.

Two shortcomings of this study are as follows: First, This study focused on the relationship between environmental strategy, performance feedback, and ESG performance, but corporate environmental governance involves numerous factors, and future research could explore other influencing factors such as corporate culture and stakeholder pressure. Second, The analysis in this paper is based on data from Chinese A-share listed companies, and future research can expand to investigate variances in ESG performance and their reasons, providing significant references for worldwide corporate environmental governance.

## Supporting information

S1 Data (XLSX)
